# Coagulation factor protein abundance in the pre-septic state predicts coagulopathic activities that arise during late-stage murine sepsis

**DOI:** 10.1016/j.ebiom.2022.103965

**Published:** 2022-03-27

**Authors:** Douglas M. Heithoff, Genaro Pimienta, Scott P. Mahan, Won Ho Yang, Dzung T. Le, John K. House, Jamey D. Marth, Jeffrey W. Smith, Michael J. Mahan

**Affiliations:** a Department of Molecular, Cellular, and Developmental Biology, University of California, Santa Barbara CA 93106, USA; b Institute for Collaborative Biotechnologies, University of California, Santa Barbara, CA 93106, USA; c Cancer Metabolism and Signaling Networks Program, Sanford Burnham Prebys Medical Discovery Institute, La Jolla, CA 92037, USA; d Department of Medical Microbiology and Immunology, School of Medicine, University of California, Davis CA 95616, USA; e Glycosylation Network Research Center and Department of Systems Biology, College of Life Science and Biotechnology, Yonsei University, Seoul 03722, Republic of Korea; f Infectious and Inflammatory Diseases Research Center, Sanford Burnham Prebys Medical Discovery Institute, La Jolla, CA 92037, USA; g Department of Pathology, University of California, La Jolla, San Diego, CA 92093, USA; h Faculty of Science, Sydney School of Veterinary Science, The University of Sydney, Camden, New South Wales 2570, Australia

**Keywords:** Sepsis, Sepsis diagnosis, Sepsis treatment, Factor XI, Coagulopathy, Matrix metalloprotease inhibitor

## Abstract

**Background:**

Although sepsis accounts for 1 in 5 deaths globally, few molecular therapies exist for this condition. The development of effective biomarkers and treatments for sepsis requires a more complete understanding of host responses and pathogenic mechanisms at early stages of disease to minimize host-driven pathology.

**Methods:**

An alternative to the current symptom-based approach used to diagnose sepsis is a precise assessment of blood proteomic changes during the onset and progression of *Salmonella* Typhimurium (*ST*) murine sepsis.

**Findings:**

A distinct pattern of coagulation factor protein abundance was identified in the pre-septic state– prior to overt disease symptoms or bacteremia– that was predictive of the dysregulation of fibrinolytic and anti-coagulant activities and resultant consumptive coagulopathy during *ST* murine sepsis. Moreover, the changes in protein abundance observed generally have the same directionality (increased or decreased abundance) reported for human sepsis. Significant overlap of *ST* coagulopathic activities was observed in Gram-negative *Escherichia coli*– but not in Gram-positive staphylococcal or pneumococcal murine sepsis models. Treatment with matrix metalloprotease inhibitors prevented aberrant inflammatory and coagulopathic activities post-*ST* infection and increased survival. Antibiotic treatment regimens initiated after specific changes arise in the plasma proteome post-*ST* infection were predictive of an increase in disease relapse and death after cessation of antibiotic treatment.

**Interpretation:**

Altered blood proteomics provides a platform to develop rapid and easy-to-perform tests to predict sepsis for early intervention via biomarker incorporation into existing blood tests prompted by patient presentation with general malaise, and to stratify Gram-negative and Gram-positive infections for appropriate treatment. Antibiotics are less effective in microbial clearance when initiated after the onset of altered blood proteomics as evidenced by increased disease relapse and death after termination of antibiotic therapy. Treatment failure is potentially due to altered bacterial / host-responses and associated increased host-driven pathology, providing insight into why delays in antibiotic administration in human sepsis are associated with increased risk for death. Delayed treatment may thus require prolonged therapy for microbial clearance despite the prevailing notion of antibiotic de-escalation and shortened courses of antibiotics to improve drug stewardship.

**Funding:**

National Institutes of Health, U.S. Army.

**Research in context**

**Evidence before this study**

Patients with sepsis continue to experience significant morbidity and mortality despite coordinated efforts by the *Third International Consensus Definitions for Sepsis and Septic Shock* (*Sepsis-3*) to align current understanding of research and patient management. In the clinical setting, sepsis is diagnosed by a symptom-based approach that may include decreased oxygen saturation, altered mental status, decreased systolic blood pressure, acidosis, abnormal kidney or liver function, decreased platelet counts and thrombosis or bleeding. A suspected infection may be confirmed by positive body fluid culture (blood, urine, cerebrospinal fluid etc.), but this may only occur at late stages of disease or not at all in a substantial fraction of cases. Biomarkers of inflammation and coagulopathy remain exceedingly rare and those identified have somewhat limited clinical utility (e.g., C-reactive protein, procalcitonin etc.). The development of effective biomarkers and treatments for sepsis thus requires a more complete understanding of host responses and pathogenic mechanisms at early stages of disease to minimize host-driven injury.

**Added value of this study**

An alternative to the current symptom-based approach for sepsis diagnosis is a precise assessment of blood proteomic changes that occur at early versus late stages of disease. We found that blood proteomic changes occurred in a closely-timed catastrophic fashion prior to overt disease symptoms or bacteremia post-*Salmonella* infection. The marked similarities of coagulopathic activities in Gram-negative– but not in Gram-positive– sepsis suggests that sepsis can be potentially stratified by different disease mechanisms elicited by discrete pathogens, dispelling the prevailing notion that sepsis manifests as a stereotypical process spanning inflammation and coagulopathy. Antibiotic treatment regimens initiated after specific changes arise in the plasma proteome were predictive of increased disease relapse and death after completion of antibiotic therapy.

**Implications of all the available evidence**

Altered blood proteomics provides a platform to develop rapid and easy-to-perform tests to forecast sepsis for early intervention via biomarker incorporation into existing blood tests prompted by patient presentation with general malaise versus overt disease symptoms, and to stratify pathogens for appropriate treatment. Clinical benefit is manifested by the acquisition of risk profile data well-before overt disease symptoms necessary for Sequential Organ Failure Assessment (SOFA) and quick SOFA (qSOFA) scores used to evaluate intensive care unit (ICU) and non-ICU patients, respectively. Time to antibiotic treatment is the principal tenet for improved patient outcome during emergency care for sepsis and is consistent with the “Surviving Sepsis Campaign” recommendations for early and aggressive antibiotics for all patients with possible sepsis or septic shock. This study demonstrates that antibiotics are less effective in microbial clearance after the onset of altered blood proteomics as evidenced by increased disease relapse and death after termination of antibiotic therapy. Treatment failure may be due to altered bacterial / host responses and associated host-driven injury, providing insight into why delays in antibiotic administration in human sepsis are associated with increased risk for death. Delayed treatment may thus require extended therapy for microbial clearance despite established physician guidance of antibiotic de-escalation and shortened courses of antibiotics to improve drug stewardship.

Alt-text: Unlabelled box

## Introduction

Sepsis is a life-threatening disease due to a dysregulated host response to infection, triggering severe inflammation, tissue coagulopathy, and organ dysfunction.^1^ Sepsis accounts for 1 in 5 deaths globally, is the most common cause of deaths in U.S. hospitals, and is more prevalent worldwide than cancer, with an estimated global annual burden of nearly 50 million cases and 11 million deaths.^2^ Even with adherence to rapid diagnosis and treatment protocols, mortality averages 25% following severe sepsis or septic shock, with many survivors experiencing lifelong physiological debilitation with cognitive decline reflecting vascular and organ damage.^3^, ^4^, ^5^ The costs of sepsis treatment are escalating and estimated to exceed $24 billion annually in the U.S.^6^ Despite this urgent medical need, few molecular therapies exist for this condition.^7^^,^^8^

Dysregulation of inflammation and coagulation during sepsis are the primary sources of host-driven pathology resulting in disability and death.^9^ The excessive presence of inflammatory cytokines in circulation and tissues during sepsis and septic shock– termed the “cytokine storm”– triggers severe systemic inflammation and resultant pathologic injury to vascular and organ systems.^10^^,^^11^ Although multiple cytokines that drive inflammatory tissue and vascular injury have been implicated, their therapeutic modulation has yet to lead to useful therapies and, in some cases, have increased patient mortality.^12^, ^13^, ^14^ Dysregulation of blood coagulation with pathologic thrombosis and hemorrhage– termed disseminated intravascular coagulation (DIC)– is the result of inflammatory cytokine-initiated coagulation coupled with dysregulation of anti-coagulant and fibrinolytic activities that lead to endothelial cell dysfunction and microvascular thrombosis.^15^^,^^16^ Once the DIC syndrome is triggered, these unrestrained activities can cause stroke, myocardial infarction, embolism, hemorrhage, and organ failure.

In the clinical setting, sepsis diagnosis is defined by the appearance of multiple physiologic and biochemical abnormalities that may include decreased oxygen saturation, altered mental status, decreased systolic blood pressure, abnormal serum creatinine, lactate, C-reactive protein (CRP) and bilirubin, decreased platelet counts and coagulopathic changes such as increased D-dimer, thrombosis, or bleeding.^17^ Biomarkers of inflammation and coagulopathy remain exceedingly rare and those identified have somewhat limited clinical utility (e.g., CRP, procalcitonin, IL-6, presepsin, CD64, lipopolysaccharide binding protein (LBP)).^7^^,^^8^^,^^18^ Currently, automated blood culture systems are the gold-standard to detect bacteremia, which are coupled to diagnostic techniques for pathogen identification.^19^ Examples include multiplex PCR (BioFire, bioMerieux), real time multiplex PCR (Xpert, Cepheid), DNA microarray (Verigene, Luminex), and fluorescent *in situ* hybridization (PNA Fish, AdvanDx). However, these techniques require positive blood cultures, detect only targeted pathogens, and are influenced by prior antibiotic exposure. Thus, the development of effective biomarkers and treatments for sepsis requires a more complete understanding of host responses and pathogenic mechanisms at early stages of disease to minimize host-driven injury.^20^

Recent approaches are beginning to reveal important insights into the role of host responses and their impact on sepsis. The Plasma Proteome Signature of Sepsis (PPSS) is a functionally-connected network initially identified as being common to five Gram-negative and Gram-positive murine models of late-stage sepsis, with similar changes in the expression of these proteins observed in human sepsis.^21^ Additionally, serum analysis of sepsis patients revealed pathogenic-proteomic-signatures for prediction of *Staphylococcus aureus* patient mortality risk profiling.^22^ However, an early detection system that does not depend on blood culture or pathogen identification, and can stratify Gram-negative vs. Gram-positive sepsis, would be immensely important to patient care due to their potential vast differences in antibiotic susceptibilities. Herein, we applied temporal investigations of blood proteome remodeling in *ST* sepsis to provide a potential means for early sepsis diagnosis before overt symptoms or bacteremia, which is not available to late-stage sepsis studies alone, wherein host responses and pathogenic mechanisms may consolidate into a stereotypical process of aberrant inflammatory and coagulopathic activities.^20^

## Methods

### Bacterial strains and culture conditions

*Salmonella enterica* serovar Typhimurium (*ST*) reference strain ATCC 14028 (CDC 6516-60), *Escherichia coli (EC)* strain ATCC 25922 (clinical isolate, FDA strain Seattle 1946), methicillin-sensitive *Staphylococcus aureus* (MSSA) strain Newman, methicillin-resistant *Staphylococcus aureus* (MRSA) strain CA-MRSA USA300, and *Streptococcus pneumoniae* (*SPN)* serotype 2 strain D39 were grown as previously described.^20^

### Bacterial infections

*ST* was administered via gastric intubation at a dose of 1 × 10^7^ colony forming units (cfu) (20X LD_50_); *EC* and *SPN* were administered i.p.; *EC* at a dose of 1 × 10^7^ cfu (10X LD_50_) and *SPN* at a dose of 1 × 10^4^ cfu (10X LD_50_); MRSA and MSSA were administered i.v.; MRSA at a dose of 1 × 10^8^ cfu (20X LD_50_) and MSSA at a dose of 5 × 10^8^ cfu (20X LD_50_).^20^ Animal experiments were carried out with 8–12-week-old C57BL/6J mice and used equal numbers of males and females. All mice in the study were provided sterile pellet food and water ad libitum.

### Sepsis time points

*ST: t* = 0 (pre-infection), *t =* 5 days (early sepsis), and *t =* 8 days (late sepsis); *EC: t =* 0 (pre-infection), *t =* 24 h (early sepsis), and *t =* 48 h (late sepsis); MSSA: *t =* 0 (pre-infection), *t =* 24 h (early sepsis), and *t =* 96 h (late sepsis); MRSA: *t =* 0 (pre-infection), *t =* 24 h (early sepsis), and *t =* 48 h (late sepsis); *SPN: t =* 0 (pre-infection), *t =* 24 h (early sepsis), and *t =* 48 h (late sepsis).^20^ For all five bacterial strains, disease severity and mortality are directly proportional to increased bacterial cfu in the bloodstream and not the bacterial dose or route of administration.^20^

### Proteomics plasma sample preparation

Proteomics samples were prepared as described previously,^21^ with the exception of the depletion step, which was optimized as described below. Albumin and immunoglobulin depletion were performed by precipitating 10 μL of plasma with pre-chilled acetone (20 °C), air-dried and incubated for an hour at 4 °C with a mix of antibodies specific to albumin (SigmaAldrich LSKMAGL10) and IgA/G (ThermoFisherScientific 88802), crosslinked to magnetic 92 beads. The eluate was collected and incubated once more with magnetic beads. Protein eluates were cysteine-reduced and alkylated with DTT and iodoacetamide, respectively, followed by overnight digestion with mass spectrometry grade trypsin protease (PIERCETM 90057). Equal amounts of protein digests from six time points (days 0, 2, 3, 4, 5, 8 post-infection) were labeled with specific isotopically-labeled tandem mass tags (TMT). Each time point included plasma from two biological replicates (two mice).

### Proteomics data collection, analysis and interpretation

TMT-labeled sample from each biological replicate was injected three times in a nano-Liquid Chromatography system (nLC) connected inline to a LUMOS-Trybrid Orbitrap tandem mass spectrometer (MS/MS). To increase the number of peptides targeted for sequencing by MS/MS fragmentation, a two-dimensional (2D) nLC prefractionation setup was used. The first dimension was a low resolution C18 reverse-phase separation at high pH that produced 12 fractions. Each fraction was in turn separated at low pH in a high resolution C8 reverse phase and ionized for MS/MS fragmentation and MS determination. The separation and data collection methods were as described previously.^21^ Data protein identification assignments and TMT ratio calculations were performed using the latest version of MaxQuant (1.6.8).^23^ Statistical calculations and plots were done in RStudio (1.1.463). To identify and quantify bacterial and host protein abundance simultaneously, we combined the most updated proteome databases from UniProtKB for *Salmonella* enterica serovar Typhimurium 14028 and the mouse strain C57BL/6J. Proteomics data is provided in Supplementary Table 1: **a**, network annotation; **b**, protein expression ratios; **c**, temporal analysis; **d**, STRING network coordinates; **e**, network calculations.

### ELISA assays

Cytokine enzyme-linked immunosorbent assay (ELISA) assays were performed and validated according to manufacturer's instructions, including IL-1β (BMS6002; Thermo Fisher Scientific), IL-6 (KMC0061; Thermo Fisher Scientific), IFNγ (KMC4021; Thermo Fisher Scientific) and TNFα (BMS607-3; Thermo Fisher Scientific).

### Coagulation factor and platelet assays

Coagulation factor assays. All clot-based coagulation assays were performed on platelet-poor citrated plasma in duplicate using an ST-4 semi-automated coagulometer (DiagnosticaStago, NJ). Chromogenic substrate-based assays were performed with SpectraMax iD3 Multi-Mode Microplate Reader (Molecular Devices, CA). The methodology of all coagulation studies were performed according to established protocols.^24^^,^^25^Platelet Assays. Approximately 20 µL of whole blood collected from mice by cardiac puncture was added to microtainer tubes with EDTA (BD Biosciences) and analyzed within 2 h of collection using a Hemavet 950 FS instrument (Drew Scientific) programmed with mouse hematology settings.^25^

### Alkaline phosphatase assay

Mice were infected via gastric intubation with *ST* (5 × 10^6^ cfu) and blood was collected into microtainer serum separator tubes (BD Biosciences) with no anticoagulant and allowed to clot for 30 m at room temperature. Serum was collected after centrifugation at 13,000 rpm for 10 m. Serum chemistry analyses, including measurements of alkaline phosphatase (AP) activity, were acquired with a VetScan Comprehensive Diagnostic Profile reagent rotor using the VetScan Chemistry Analyzer as previously reported.^20^

### Neuraminidase assay

Mice were infected via gastric intubation with *ST* (5 × 10^6^ cfu) and blood was collected into K_2_ EDTA microtainer tubes (BD Biosciences) and mixed on a rotator for 10 m at room temperature. Plasma was collected after centrifugation at 13,000 rpm for 10 m. Neuraminidase (Neu) activity was detected via Amplex Red (Thermo Fisher Scientific) using 25 µL plasma as per manufacturer recommendations. Control Neu activity was determined using a neuraminidase standard (*Arthrobacter ureafaciens*; EY Laboratories) serially diluted 2-fold in PBS starting at 500 U/L.

### Matrix metalloproteinase inhibitor treatment

Mice were infected via gastric intubation with *ST* (5 × 10^6^ cfu). At 2 h post-infection, mice were treated with MMP inhibitor, GM6001 (inhibits MMP 1/2/3/8/9; CC1100, Millipore Sigma Aldrich),^26^ administered i.p. at a dose of 200 µg per mouse per day for 8 days; vehicle control mice were administered GM6001 diluent on the same treatment regimen.

### Antibiotic treatment

Mice infected via gastric intubation with *ST* (10^7^ cfu) were treated with the following dosing regimens: ceftriaxone (50 mg/kg/day) or ciprofloxacin (30 mg/kg/day) were administered every 12 h via the intraperitoneal route of administration on the days indicated.

### Ethics

All animal experimentation was conducted following the National Institutes of Health guidelines for housing and care of laboratory animals and performed in accordance with Institutional regulations after pertinent review and approval by the Institutional Animal Care and Use Committees at the University of California, Santa Barbara (#445) and Sanford Burnham Prebys Medical Discovery Institute (#A3053) (La Jolla, CA).

### Statistics

Student's unpaired t-test was used to compare the means of two groups for cytokine and platelet analyses; GraphPad Prism software version 8.0 was used to determine statistical significance determined by unpaired 2-tailed t-test. Log-rank (Mantel-Cox) test was used to compare differences in survival between groups for Kaplan-Meier survival curves; significance was determined using GraphPad Prism version 9.0. *P* values of less than 0.05 were considered significant. The exact value of n, representing the number of mice, was indicated in the figure legends. Proteomic responses to infection were screened according to the ratio of the amount of each protein following infection relative to pre-infection (Supplementary Table 1b) as described previously.^21^  A two-sample *t*-test was calculated, based on three technical replicates (injections) per sample in control and sepsis samples. Inflammatory and coagulopathic proteins with changes in the ratio of sepsis/control (SE/CTL) of >0.6 (log2)/1.5-fold, (natural number), with *P* values < 0.1 selected to be included in the subsequent assessment of activity experiment. The statistical analysis of Tables 1 and 2 as well as the protein expression ratios in Supplementary Table 1b was conducted utilizing RStudio.^27^ As the data was not normally distributed, the distribution of the data was analyzed using Permutational Multivariate Analysis of Variance utilizing the vegan package.^28^ Table 1: Pathogen and time were included as independent variables and the results of each analyte as dependent variables. Time, pathogen and the interaction between time and pathogen were significant (*P* < 0.05). Pairwise comparisons were performed between uninfected mice (*n* = 60) and early (*n =* 12) and late (*n =* 12) septic mice for each analyte and pathogen. Benjamini-Hochberg was utilized to adjust *P* values for multiple comparisons. Table 2: Time and treatment were included as independent variables and the results of each analyte as dependent variables. Time, treatment and the interaction between time and treatment were significant (*P* < 0.05). Pairwise comparisons were performed between the untreated and the corresponding treated group for each analyte (early sepsis: untreated vs. treated; late sepsis: untreated vs. treated). Benjamini-Hochberg was utilized to adjust *P* values for multiple comparisons. *n =* 15 mice per group.

**Table 1:** Temporal analysis of coagulation factor activities in mice infected with Gram-negative and Gram-positive pathogens.

	Not infected	*S.* Typhimurium		*E. coli*		MSSA		MRSA		*S. pneumoniae*	
Analyte (% NMP)		Early sepsis (Day 5)	Late sepsis (Day 8)	Early sepsis (24 h)	Late sepsis (48 h)	Early sepsis (24 h)	Late sepsis (96 h)	Early sepsis (24 h)	Late sepsis (48 h)	Early sepsis (24 h)	Late sepsis (48 h)
Antithrombin	142	134	108*	108*	91*	104*	123	120	109*	143	79*
Protein C	106	90	87	107	100	85	172*	135*	131	82*	76*
Protein S	97	84	131*	210*	171*	181*	131*	193*	221*	93	151*
α-2 antiplasmin	165	167	159	170	146	158	246*	200	224*	173	153
Fibrinogen (mg/dL)	218	292*	383*	577*	429*	675*	839*	720*	964*	325*	399*
Factor II	87	97*	99*	123*	98*	118*	179*	129*	131*	79*	65*
Factor V	75	82	107*	151*	127*	109*	189*	129*	128*	68	60*
Factor VII	73	78	92*	102*	79	94*	157*	118*	114*	60*	61*
Factor VIII	76	78	64	72	63	89	190*	100*	119*	63	49*
Factor IX	82	76	84	79	69	74	166*	101*	120*	67*	54*
Factor X	91	89	74*	107*	91	87	117*	111*	118*	77*	58*
Factor XI	66	82*	190*	78*	86*	91*	180*	89*	110*	51*	81*
Factor XII	95	81*	77*	88	88	54*	64*	74*	66*	103*	92
vWF	100	82	121	230*	255*	239*	389*	220*	328*	109	108

Mice were infected with *S*. Typhimurium, *E. coli*, MSSA, MRSA or *S. pneumoniae* as described in the methods and plasma was collected at the indicated times and analyzed for coagulation activities. Mean % values are relative to mouse normal pooled plasma (NMP). Pathogen and time were included as independent variables and the results of each analyte as dependent variables. Time, pathogen and the interaction between time and pathogen were significant (*P < 0.05). Pairwise comparisons were performed between uninfected mice (*n* = 60) and early (*n* = 12) and late (*n* = 12) septic mice for each analyte and pathogen. Benjamini-Hochberg was utilized to adjust *P* values for multiple comparisons.

**Table 2:** Effect of MMP inhibitor GM6001 on coagulation factor activities during *S.* Typhimurium sepsis.

*S.* Typhimurium	− GM6001			+ GM6001		
Analyte (% NMP)	Not infected	Early sepsis (Day 5)	Late sepsis (Day 8)	Not infected	Early sepsis (Day 5)	Late sepsis (Day 8)
Fibrinogen (mg/dL)	189	227	351	152	261	226*
Factor II	76	67	46	73	73	76*
Factor V	83	80	60	70	80	92*
Factor VII	93	90	105	81	104	111
Factor VIII	72	74	53	58	94	72
Factor IX	70	64	90	52*	60	60*
Factor X	87	81	45	82	82	75*
Factor XI	72	71	260	62	51*	70*
Factor XII	84	78	61	77	66	64

Mice were orally infected with *S.* Typhimurium (10^7^ cfus) and treated in the presence/absence of MMP inhibitor, GM6001. Plasma was collected at the indicated times and analyzed for coagulation activities. % values are relative to mouse normal pooled plasma (NMP). Time and treatment were included as independent variables and the results of each analyte as dependent variables. Time, treatment and the interaction between time and treatment were significant (*P < 0.05). Pairwise comparisons were performed between the untreated and the corresponding treated group for each analyte (early sepsis: untreated vs. treated; late sepsis: untreated vs. treated). Benjamini-Hochberg was utilized to adjust *P* values for multiple comparisons. *n* = 15 mice per group.

### Role of funders

Funders had no role in the study design, data collection, data analyses, interpretation, or writing of the report.

## Results

### A distinct pattern of coagulation factor protein abundance in the pre-septic state was predictive of coagulopathic activities during late-stage disease

**Figure 1:**
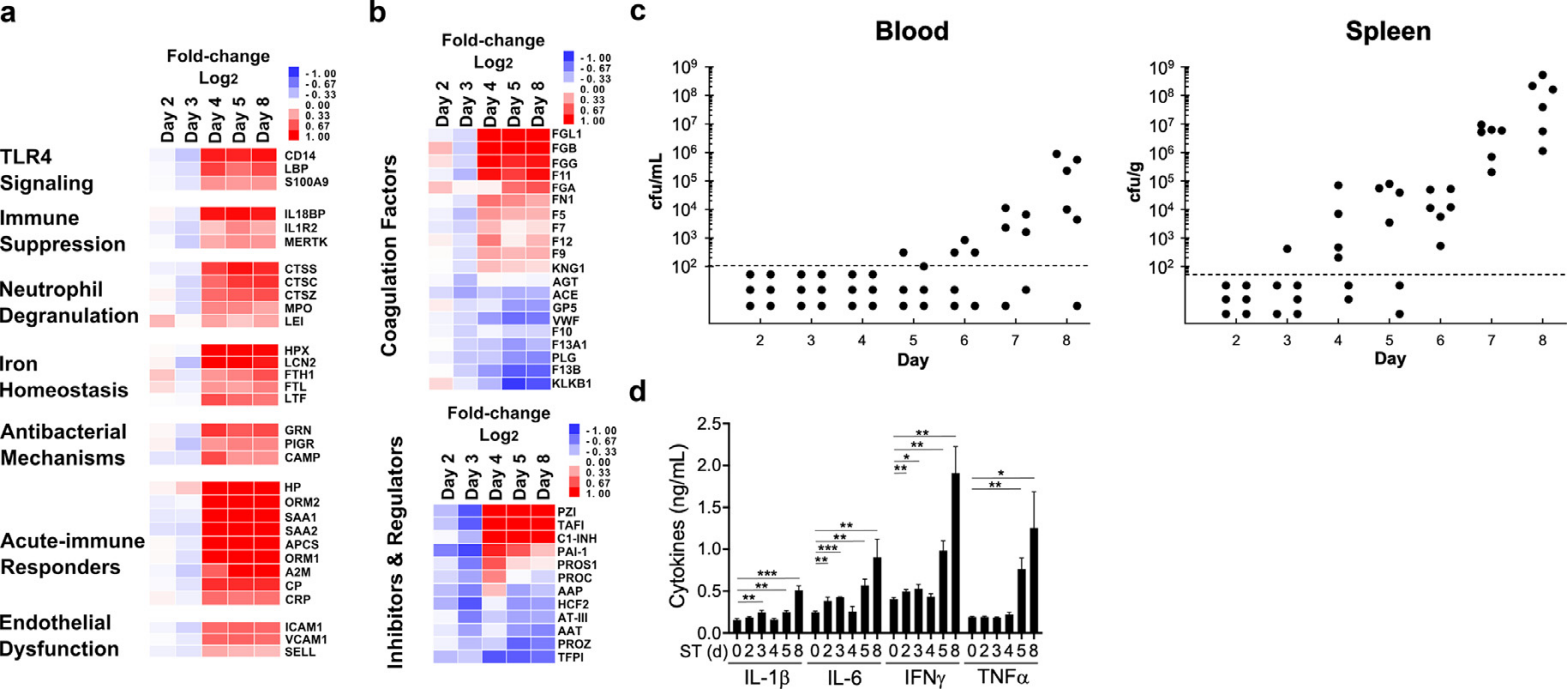
**Temporal changes to the plasma proteome, cfus in circulation, and innate immune cytokines following *S.* Typhimurium infection.** Plasma proteomics was determined by MS analysis of samples collected from mice at five time points post-*S.* Typhimurium (*ST*) infection (day 0, 2, 3, 4, 5, 8). Heatmap representation of the fold-change (Log_2_) of (**a**) functional sub-networks and (**b**) coagulation pathways upon unsupervised hierarchical clustering (*n* = 2 mice). Supporting proteomics data is provided in Supplementary Table 1: a, network annotation; b, protein expression ratios; c, temporal analysis; d, STRING network coordinates; e, network calculations. **c**, Mice were infected with *ST* and assessed for cfus in circulation and spleen (*n* = 6 mice). Limit of detection (LOD) = 100 cfu/mL blood; 50 cfu/gram spleen. **d**, Matched samples from the plasma proteome analysis were assessed for innate immune cytokine analysis via ELISA (*d* = days; *n* = 3 mice/time point). *P < 0.05; **P < 0.01; ***P < 0.001.

Plasma proteomics was determined at five time points post-*ST* infection (day 0, 2, 3, 4, 5, 8). Early-onset remodeling of the plasma proteome occurred in a narrowly-timed, catastrophic fashion by day 4 post-infection–prior to onset of overt disease symptoms or bacteremia– and correlated with the onset of tissue colonization (Figure 1a–c). Examples include the early-onset detection of the acute-phase immune response proteins, CRP and LBP, which are benchmark biomarkers for human sepsis^7^^,^^8^^,^^18^; and increased abundance of immune modulator proteins CD14 and myeloperoxidase (MPO) that stimulate TLR4 and neutrophil activation^29^^,^^30^ (Figure 1a). As expected, such changes ultimately led to proinflammatory cytokine production during late-stage sepsis (IL-1β, IL-6, IFNγ, TNFα) (Figure 1d). Further, marked overlap was observed with recently-discovered proteomic-signatures of Gram-positive *S. aureus* patient mortality, sharing 14 of the top 25 biomarkers, including decreased expression of anticoagulant heparin cofactor 2 (HCF2) and increased expression of hemostasis proteins fibronectin and proteoglycan 4 (PRG4).^22^ However, immensely important to patient care are early-onset changes that are not available to late-sepsis studies alone wherein host responses and pathogenic mechanisms may consolidate into stereotypical processes of aberrant inflammatory and coagulopathic activities.

To this end, we identified 119 plasma proteins that exhibited a significant change in expression relative to uninfected animals, including specific time-dependent functional interactions that occur at early versus late stages of disease (**Supplementary Figure 1; Supplementary Table 1a–e**).^20^ A distinct pattern of coagulation factor protein abundance in the pre-septic state was identified including factor XI (FXI) and fibrinogen subunits (FGA, FGB, FGG), concomitant with a progressive reduction in abundance of protease inhibitors antithrombin-III (AT-III), HCF2, and alpha-1-antitrypsin (AAT) (Figure 1b). Further, the changes in protein abundance observed in *ST* murine sepsis generally have the same directionality (increased or decreased abundance) as reported in the literature for human sepsis (**Supplementary Table 1b,c)**.^21^ Notably, this distinct pattern of coagulation factor abundance was predictive of coagulopathic activities that occurred during late-stage disease. Examples include increased fibrinogen as well as increased protein S, FII, FV, FVII, and FXI activities, coupled with diminished levels of AT-III, FX, and FXII activities during *ST* sepsis (Table 1).

### Gram-negative infections elicited similar changes of coagulopathic activities during sepsis

**Figure 2:**
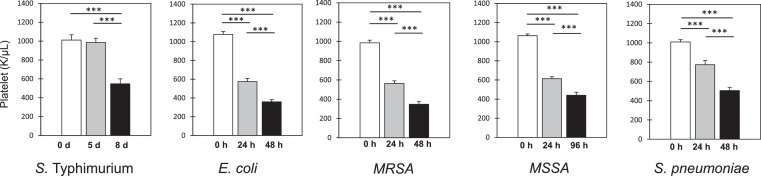
**Temporal changes to platelet abundance following infection with Gram-negative and Gram-positive organisms*.*** Mice were infected with *S.* Typhimurium (gastric intubation; 10^7^ cfu), *E. coli* (i.p.; 10^7^ cfu), MSSA (i.v.; 5 × 10^8^ cfu), MRSA (i.v.; 10^8^ cfu) or *S. pneumoniae* (i.p.; 10^4^ cfu) and blood was assessed for platelet abundance via Hemavet analysis. *n* = 16 mice/cohort. Data are presented as means ± SEM (***P < 0.001).

Significant overlap of *ST* coagulopathic activities was observed in Gram-negative *E. coli* sepsis, but not in Gram-positive sepsis models studied herein. Examples include increased fibrinogen as well as increased protein S, FII, FV, and FXI activities, while exhibiting diminished levels of AT-III activity (Table 1). In contrast, Gram-positive organisms, including methicillin-sensitive (MSSA) and methicillin-resistant (MRSA) *S. aureus*, elicited a general pattern of increased coagulation factor activation (e.g., FII, FV, FVII – XI), while *S. pneumoniae* elicited a specific pattern of decreased coagulation factor activation (e.g., FII, FV, FVII – FX). Additionally, contrary to the specific coagulation factor changes observed during Gram-negative sepsis, a shared moderate thrombocytopenia was elicited by all Gram-negative and Gram-positive organisms tested herein (Figure 2).

### Treatment with matrix metalloprotease inhibitors prevented aberrant inflammatory and coagulopathic activities and increased the frequency of survival post *ST* infection

**Figure 3:**
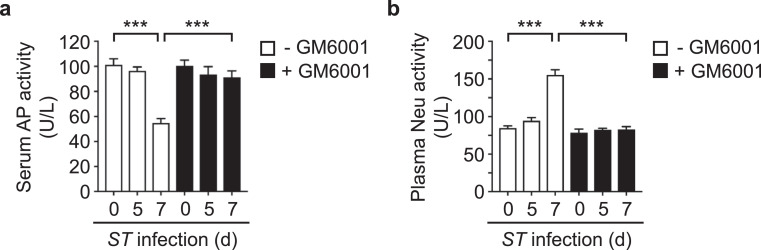
**Effect of MMP inhibitor (GM6001) treatment on host alkaline phosphatase and neuraminidase activities during *S.* Typhimurium sepsis.** Mice were infected via gastric intubation with *S.* Typhimurium (*ST*) (5 × 10^6^ cfu). At 2 h post-infection, mice were treated in the presence and absence of GM6001, administered i.p. at a dose of 200 µg per mouse per day for 8 days; vehicle control mice were administered GM6001 diluent on the same treatment regimen. Mice were assessed for (**a**) serum AP activity (*n* = 10 mice per condition) and (**b**) plasma Neu activity (*n* = 6 mice per condition). Data are presented as means ± SEM (***P < 0.001).

**Figure 4:**
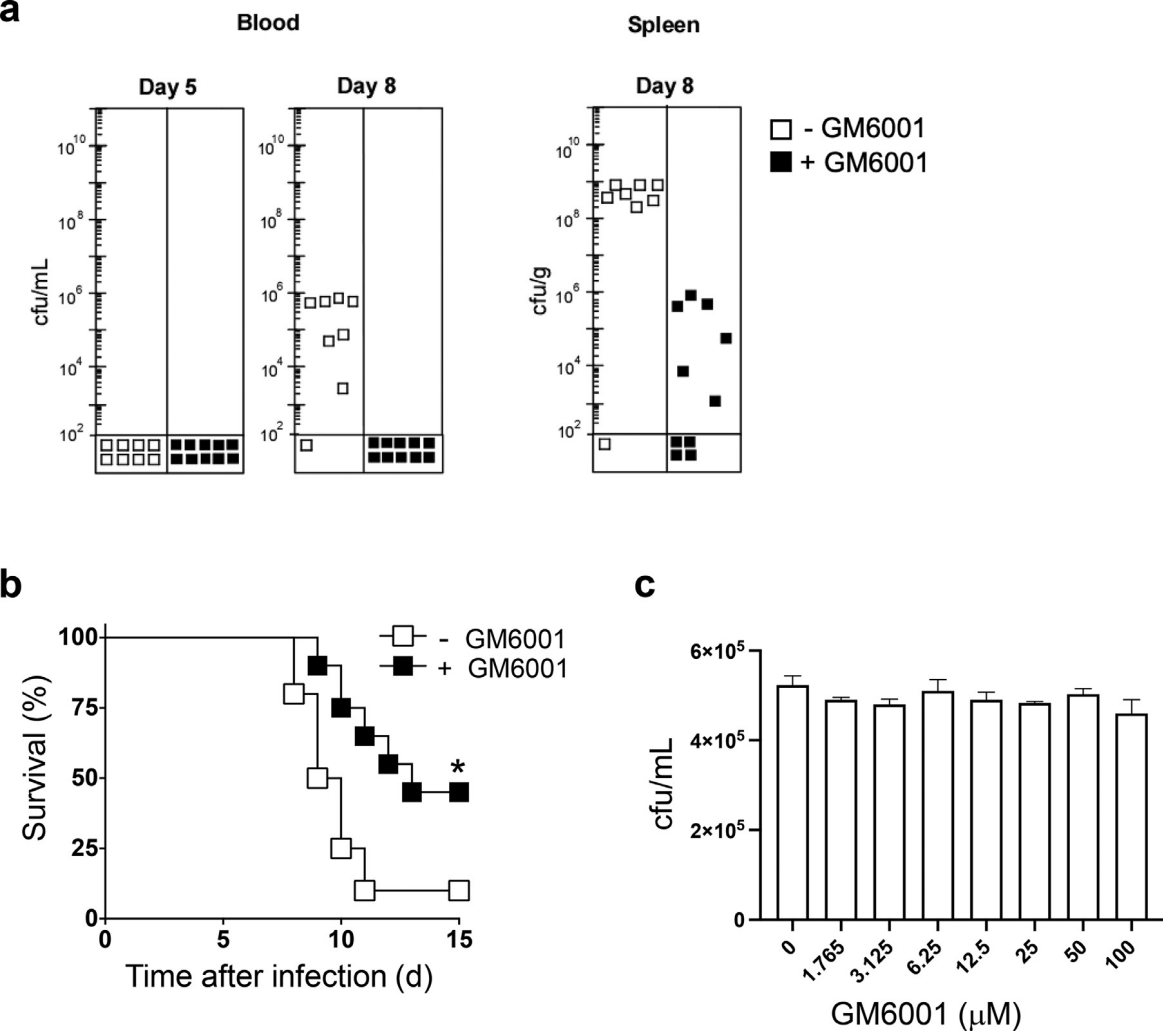
**Effect of MMP inhibitor treatment on bacterial cfus and survival post-*S.* Typhimurium infection.** Mice were infected via gastric intubation with *S.* Typhimurium (5 × 10^6^ cfu). At 2 h post-infection, mice were treated in the presence and absence of MMP inhibitor, GM6001, administered i.p. at a dose of 200 µg per mouse per day for 8 days; vehicle control mice were administered GM6001 diluent on the same treatment regimen. Mice were assessed for (**a**) cfus in circulation and spleen (*n* = 8–10 mice/cohort) and (**b**) mouse survival (*n* = 20 mice/cohort). *P <0.05. **c**, *ST* was co-incubated in the presence/absence of GM6001 *in vitro* and assessed for viability.

Host matrix-metalloproteinases (MMPs) play important roles in endothelial cell function, inflammation and coagulation.^31^, ^32^, ^33^ Thus, we questioned whether the administration of the MMP inhibitor, GM6001,^26^ could prevent aberrant inflammatory and coagulopathic activities post-*ST* infection. Daily intraperitoneal administration of GM6001 prevented the induction of host neuraminidase (Neu) activity and associated desialylation and accelerated clearance of host anti-inflammatory alkaline phosphatase (AP) activity involved in LPS detoxification (Figure 3a,b).^20^ The presence of MMP inhibitor also reversed coagulopathic changes as evidenced by blocking the induction of fibrinogen and FXI activity and preventing consumption of multiple coagulation factors (e.g., FII, FV, FIX, FX) (Table 2). Additionally, the presence of GM6001 reduced bacterial cfus in circulation and spleen (Figure 4a), and improved mouse survival post-*ST* infection (Figure 4b). Co-incubation of GM6001 with *ST in vitro* had no effect on bacterial viability or proliferation, which is consistent with GM6001 therapeutic effects acting through host molecular targets vs. direct action on the bacterium (Figure 4c).

### Antibiotic treatment regimens initiated after specific changes arise in the plasma proteome were predictive of increased disease relapse and death after cessation of antibiotic treatment

**Figure 5:**
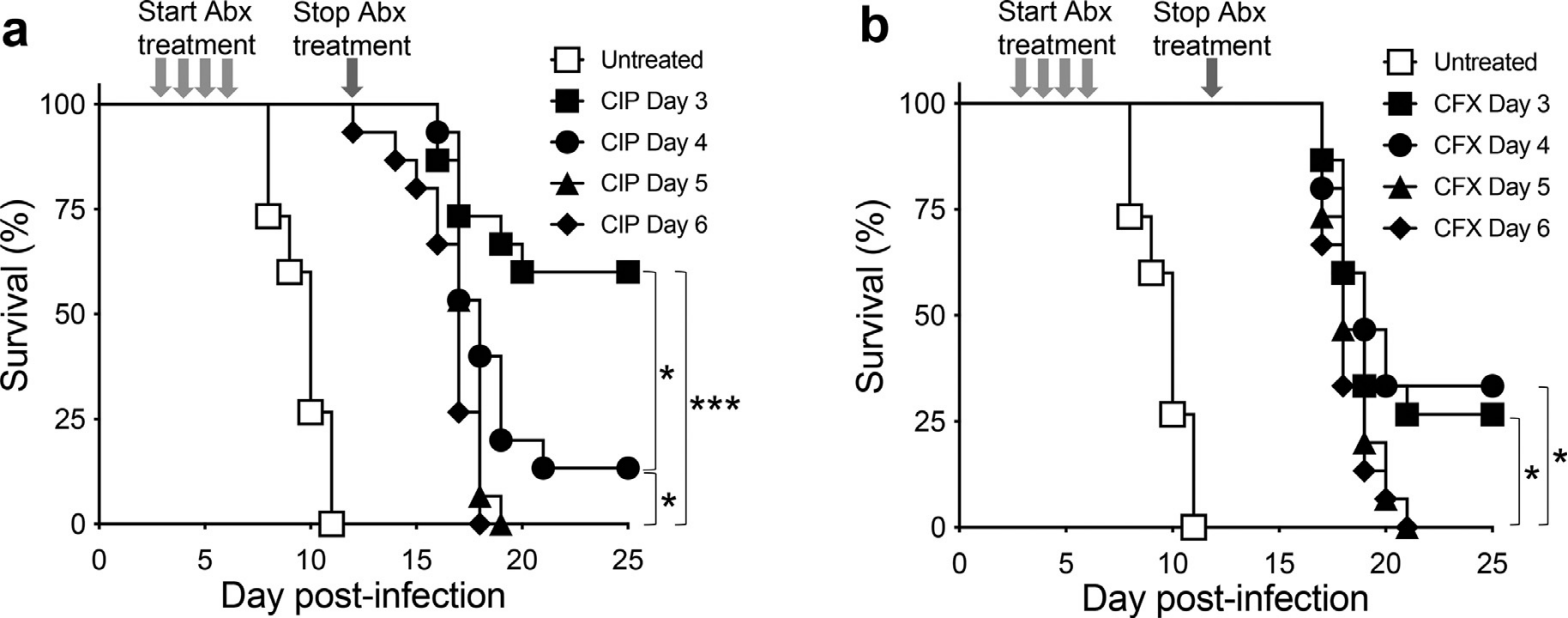
**Timing of initiation of antibiotic treatment and resultant disease relapse and death after cessation of antibiotics**. Mice were infected via gastric intubation with *S.* Typhimurium (10^7^ cfu) and treated twice daily with (**a**) ciprofloxacin (CIP; 30 mg/kg/day) or (**b**) ceftriaxone (CFX; 50 mg/kg/day), starting on either day 3, 4, 5, or 6 (green arrows) and continuing through day 12 post-infection, after which antibiotics were removed at day 13 (red arrow). Survival was monitored up to 25 d post-infection. *n* = 15 mice/cohort. *P <0.05; ***P <0.001.

Antibiotic treatment was initiated before and after blood proteome remodeling and presence of cfus in circulation. As expected, daily treatment with ciprofloxacin (Figure 5a) or ceftriaxone (Figure 5b) initiated at day 3, 4, 5, or 6 post *ST*-infection effectively cleared cfus from circulation in nearly all mice by day 8 (119/120 mice; **Supplementary Table 2**). Such treatment resulted in little-to-no overt disease symptoms through day 12 when treatment was ceased in all mouse cohorts. In comparison, 12/15 untreated mice showed high blood cfus at day 8 (>10^2^–10^7^ cfus/mL), with all (15/15) untreated mice succumbing to infection between days 8 and 11. Thus, as observed in the clinical setting,^34^^,^^35^ antibiotic treatment was effective in promoting bacterial clearance and survival even when initiated later in the disease process (day 5, 6), despite extensive blood proteome remodeling and cfus in circulation and/or spleen at the time of treatment (Figure 1a–c).

Next, we assessed whether antibiotic treatment regimens initiated after specific changes arise in the plasma proteome were predictive of increased disease relapse and death after cessation of antibiotic treatment. Antibiotics were removed at day 13 in all treated cohorts. Ciprofloxacin treatment initiated on day 3 was significantly more effective at preventing disease relapse and death relative to regimens that initiated treatment later post-infection (day 3 vs. 4, 5, 6) (Figure 5a). Notably, day 3 precedes blood proteome remodeling, with nearly all mice exhibiting below-detectable levels of cfus in the spleen (5/6 mice) and in circulation (6/6 mice) when antibiotic treatment was initiated (Figure 1a–c). Nevertheless, ∼40% of mice which initiated treatment on day 3 succumbed to infection after removal of ciprofloxacin presumably due to low-level cfus seeding antibiotic-privileged tissues such as the gallbladder.^36^ A similar trend was observed following ceftriaxone treatment (day 3, 4 vs. 5, 6) (Figure 5b). These data indicate that antibiotic treatment regimens initiated after specific changes arise in the plasma proteome were predictive of increased disease relapse and death after termination of antibiotic therapy.

## Discussion

An alternative to the current symptom-based approach used to diagnose sepsis is a precise assessment of blood proteomic changes during the onset and progression of sepsis to identify biomarkers at early stages of disease to minimize host-driven pathology. Herein, we identified a distinct pattern of coagulation factor protein abundance in the pre-septic state– prior to overt disease symptoms or bacteremia– that was predictive of the dysregulation of fibrinolytic and anti-coagulant activities and resultant consumptive coagulopathy during *ST* sepsis. Significant overlap of *ST* coagulopathic activities was observed in Gram-negative *EC* sepsis but not among Gram-positive sepsis models studied herein. Sepsis can thus be potentially stratified by different disease mechanisms elicited by discrete pathogens, dispelling the prevailing notion that sepsis manifests as a stereotypical process spanning inflammation and coagulopathy. MMP inhibitor therapeutic intervention prevented aberrant inflammatory and coagulopathic activities, reduced microbial cfus in blood and tissues, and improved survival post-*ST* infection. Antibiotic treatment regimens initiated after blood proteomic changes were predictive of an increase in disease relapse and death after cessation of antibiotics, and may reflect inadequate bacterial clearance associated with increased host-driven pathology. Such treatment failure provides a rationale as to why delays in antibiotic administration in human sepsis are associated with increased risk for death.

Marked changes to the blood proteome occurred prior to bacteremia, suggesting that tissue colonization and resultant pathogen-associated molecular patterns (PAMPs) and/or damage associated molecular patterns (DAMPs) trigger copious changes to the blood proteome at early stages post-*ST* infection.^37^ Such altered blood proteomics provides a platform to develop rapid and easy-to-perform tests to forecast sepsis for early intervention via biomarker panels that can be incorporated into existing blood tests prompted by patient presentation with general malaise and to stratify Gram-negative and Gram-positive infections for appropriate treatment. Clinical benefit is evident by the acquisition of risk profile data well-before overt disease symptoms that are necessary for SOFA and qSOFA scores used to evaluate ICU and non-ICU patients, respectively.^38^

Validation of this approach was established by the early-onset detection of the acute-phase immune response proteins, CRP and LBP, which are benchmark biomarkers for human sepsis;^7^^,^^8^^,^^18^ early-onset increased abundance of immune modulator proteins CD14 and MPO that stimulate TLR4 and neutrophil activation;^29^^,^^30^ and marked overlap of proteomic changes with recently discovered pathogenic-proteomic-signatures for prediction of *SA* patient mortality.^22^ Additionally, blood from sepsis patients has been used for (*i*) the development of DNA-based tests to identify microbes and their resistance, (*ii*) host-response RNA, (*iii*) novel biomarkers and rapid tests that detail risk profiles^39^; and (*iv*) the discovery that the source of infection contributes to the heterogeneity of the host response.^40^

A distinct pattern of coagulation factor protein abundance in the pre-septic state was identified including FXI and fibrinogen subunits, concomitant with a progressive reduction in abundance of protease inhibitors AT-III, HCF2, and AAT. Moreover, the changes in protein abundance observed in *ST* murine sepsis generally have the same directionality (increased or decreased abundance) as reported in the literature for human sepsis. Additionally, coagulation factor abundance was predictive of coagulopathic activities that occurred during *ST* sepsis; e.g., increased fibrinogen as well as increased protein S, FII, FV, FVII, and FXI activities, coupled with diminished levels of AT-III, FX, and FXII activities. Significant overlap of *ST* coagulopathic activities was observed in Gram-negative *E. coli* sepsis. In contrast, Gram-positive organisms, including MSSA and MRSA elicited a general pattern of increased coagulation factor activation, while *S. pneumoniae* elicited a specific pattern of decreased coagulation factor activation. These data suggest that early-onset coagulation protein abundance is predictive of the dysregulation of fibrinolytic and anti-coagulant activities and resultant consumptive coagulopathy during Gram-negative sepsis. FXI plays a principal role in coagulopathy as its activation promotes thrombosis,^41^^,^^42^ is detrimental to the host in several experimental sepsis models,^43^^,^^44^ and has been explored as a target for inhibition in sepsis.^45^^,^^46^

Reduced platelet abundance is a key component of outcomes in Gram-positive sepsis as platelets contribute to both host thrombosis and microbial killing activity and thus the importance of platelet abundance and its regulation in the pathogenesis of sepsis can be protective or deleterious to the host depending upon the microbial source of infection.^47^, ^48^, ^49^ Contrary to the specific coagulopathic changes observed during Gram-negative sepsis, we show that a moderate thrombocytopenia is a shared response among all five Gram-negative and Gram-positive pathogens tested herein. A moderate thrombocytopenia has been previously linked to Gram-positive sepsis due to the desialylation of platelets by either the pathogen or the host in early sepsis followed by platelet clearance involving the hepatic Ashwell-Morell receptor (AMR).^49^, ^50^, ^51^

MMPs are key regulators of the inflammation/coagulation response and are potential targets for diagnostics and therapeutics.^33^ MMPs are increased > 8-fold in the circulation of sepsis patients and are correlated with increased mortality.^31^^,^^52^^,^^53^ Additionally, MMP activity is increased > 4-fold in circulation in a murine sepsis model^31^; and MMP inhibitors protect against vascular hyporeactivity in models of acute endotoxemia in rats.^54^^,^^55^ Herein, administration of GM6001 prevented increased bacterial cfus in circulation and spleen and improved mouse survival post-*ST* infection. Such therapeutic intervention is due, in part, to blocking the induction of host Neu activity, which facilitates clearance of desialylated anti-inflammatory AP enzymes by the hepatic AMR receptor,^20^ thereby restoring the host-protective mechanism of LPS de-toxification by circulating AP enzymes. Clinical significance is evidenced by human trials indicating that AP administration reduced anti-inflammatory effects in sepsis and ulcerative colitis.^56^, ^57^, ^58^ GM6001 administration also reversed coagulopathic changes as evidenced by blocking the induction of fibrinogen and FXI activity and preventing consumption of multiple coagulation factors (e.g., FII, FV, FIX, FX). Co-incubation of GM6001 with *ST in vitro* had no effect on bacterial viability or proliferation, suggesting the therapeutic effects act through host molecular targets. Our findings further support the hypothesis that investigation into the contribution of MMPs to sepsis pathogenesis may lead to new sepsis diagnostics and therapies.

Time to antibiotic treatment is the principal tenet for improved patient outcome during emergency care for sepsis,^34^ and is consistent with the “Surviving Sepsis Campaign” recommendations for early and aggressive antibiotics for all patients with possible sepsis or septic shock.^35^ However, diagnostic uncertainty due to the current symptom-based approach used to diagnose sepsis runs the risk of damage to uninfected-but-suspected patients and virally-infected patients, who are subjected to antibiotic-related risks without any of the benefits.^59^ This study demonstrates that antibiotics are less effective in microbial clearance after the onset of altered blood proteomics as evidenced by increased disease relapse and death after termination of antibiotic therapy. Treatment failure may be due to altered bacterial / host-responses commensurate with increased host-driven pathology, providing insight into why delays in antibiotic administration in human sepsis are associated with increased risk for death. Delayed treatment may thus require prolonged duration for microbial clearance despite the prevailing notion of antibiotic de-escalation and shortened courses of antibiotics to improve drug stewardship.^60^

### Data sharing statement

Proteomics data deposition. LUMOS-Orbitrap RAW datasets and the results output from the search engine MaxQuant have been deposited in the MassIVE proteomics repository at UCSD and given the identification code PXD021291 at: https://massive.ucsd.edu/ProteoSAFe/dataset.jsp?task=1dc72a3bb34a43998f64505d209 29b61ba3. All data generated or analyzed during this study are included in this published article or in the supplement.

### Contributors

Experiments were conducted by D.M.H., G.P., S.P.M., and W.H.Y. Data were analyzed by D.M.H., G.P., S.P.M., D.T.L., J.K.H., J.D.M., J.W.S. and M.J.M. The manuscript was prepared by D.M.H., S.P.M., and M.J.M. The study was planned and directed by D.M.H., G.P., S.P.M., J.W.S., and M.J.M. All authors had full access to all data in the study and had final responsibility for the decision to submit for publication.

## Declaration of interests

The authors declare no competing interests.

## Appendix Supplementary materials

Image, application 1

Image, application 2
