# Supplementary material for: Coagulation factor protein abundance in the pre-septic state predicts coagulopathic activities that arise during late-stage murine sepsis
Source: eBioMedicine. 2022 Mar 27;78:103965. doi: 10.1016/j.ebiom.2022.103965 (PMC8965145; doi:10.1016/j.ebiom.2022.103965)
Supplement: Supplementary file 1 [file mmc1.docx]

**Supplementary Online Content**

**Heithoff, DM, Pimienta, G, Mahan, SP et al. “*Coagulation Factor Protein Abundance in the Pre-septic State Predicts Coagulopathic Activities that Arise During Late-stage Sepsis*.” *EbioMedicine.***

**Table of Contents**

1. Supplementary Figure 1. Temporal PPSS network for *ST* infection showing functional and

physical interactions.........................................................................................................................page 2

2. Supplementary Table 1. Temporal PPSS network for *S*. Typhimurium infection...........................Supplementary data #2

Supplementary Data Table of Contents

Supplementary Table 1a. Network annotation

Supplementary Table 1b. Protein expression ratios

Supplementary Table 1c. Temporal analysis

Supplementary Table 1d. STRING network coordinates

Supplementary Table 1e. Network calculations

3. Supplementary Table 2. Timing of antibiotic initiation and blood clearance.................................page 3


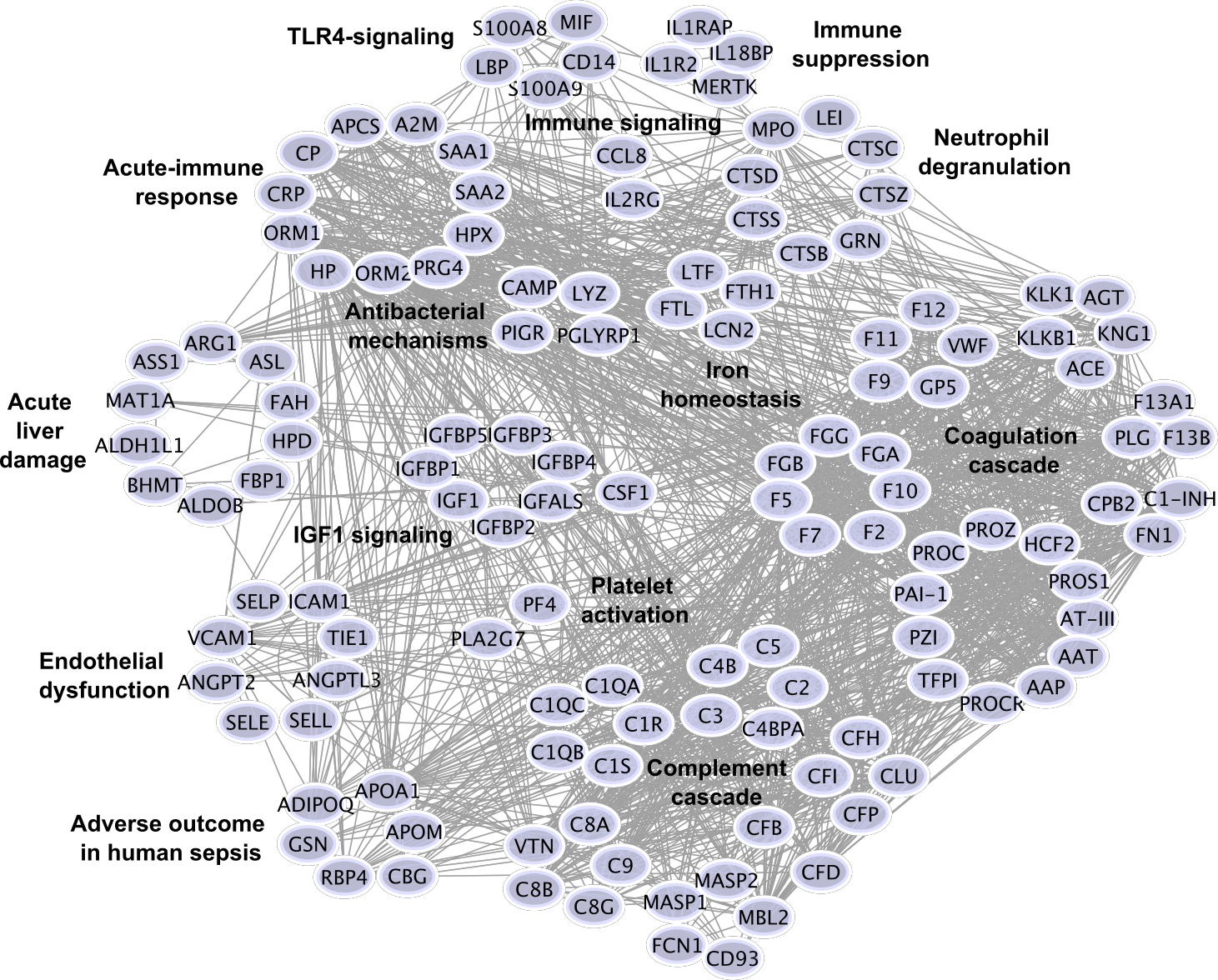


**Supplementary Figure 1.** **Temporal PPSS network for *ST* infection showing functional and physical interactions**. The temporal PPSS network for *S.* Typhimurium *(ST)* infection was integrated in STRING using default parameters and the output used to display in CYTOSCAPE for manual arrangement of functional subnetworks. Lines connecting proteins are functional or direct physical associations. Network construction analyses are provided in Supplementary Tables 1a-e.

| **Supplementary Table 2.** Timing of antibiotic initiation and bacterial clearance. | | | | | | | | | | | |
| --- | --- | --- | --- | --- | --- | --- | --- | --- | --- | --- | --- |
|  | Untreated |  | Day of Treatment Initiation | | | | | | | | |
| Mouse |  |  | CIP | | | |  | CFX | | | |
|  |  |  | Day 3 | Day 4 | Day 5 | Day 6 |  | Day 3 | Day 4 | Day 5 | Day 6 |
| 1 | 8.5 x 10^6^ |  | ND | ND | ND | ND |  | ND | ND | ND | ND |
| 2 | 8.1 x 10^6^ |  | ND | ND | ND | 1.4 x 10^3^ |  | ND | ND | ND | ND |
| 3 | 1.2 x 10^4^ |  | ND | ND | ND | ND |  | ND | ND | ND | ND |
| 4 | ND |  | ND | ND | ND | ND |  | ND | ND | ND | ND |
| 5 | 3.6 x 10^4^ |  | ND | ND | ND | ND |  | ND | ND | ND | ND |
| 6 | 1.0 x 10^7^ |  | ND | ND | ND | ND |  | ND | ND | ND | ND |
| 7 | 1.4 x 10^7^ |  | ND | ND | ND | ND |  | ND | ND | ND | ND |
| 8 | 5.0 x 10^2^ |  | ND | ND | ND | ND |  | ND | ND | ND | ND |
| 9 | 4.0 x 10^2^ |  | ND | ND | ND | ND |  | ND | ND | ND | ND |
| 10 | 4.0 x 10^3^ |  | ND | ND | ND | ND |  | ND | ND | ND | ND |
| 11 | 7.0 x 10^2^ |  | ND | ND | ND | ND |  | ND | ND | ND | ND |
| 12 | ND |  | ND | ND | ND | ND |  | ND | ND | ND | ND |
| 13 | ND |  | ND | ND | ND | ND |  | ND | ND | ND | ND |
| 14 | 1.6 x 10^4^ |  | ND | ND | ND | ND |  | ND | ND | ND | ND |
| 15 | 2.0 x 10^2^ |  | ND | ND | ND | ND |  | ND | ND | ND | ND |
| Mice were infected via gastric intubation with *S.* Typhimurium (10^7^ cfu) and either untreated, or treated with ciprofloxacin (CIP; 30 mg/kg/day) or ceftriaxone (CFX; 50 mg/kg/day) starting on either day 3, 4, 5, or 6 and continuing through day 12 post-infection. Blood cfu/mL was assessed on day 8 post-infection from the tail vein by direct colony count. n=15 mice/cohort. ND= not detectable. Limit of detection=100 cfu/mL. | | | | | | | | | | | |
